# Traumatic Brain Injury by Weight-Drop Method Causes Transient Amyloid-*β* Deposition and Acute Cognitive Deficits in Mice

**DOI:** 10.1155/2019/3248519

**Published:** 2019-03-03

**Authors:** Hajime Shishido, Masaki Ueno, Kana Sato, Masahisa Matsumura, Yasunori Toyota, Yutaka Kirino, Takashi Tamiya, Nobuyuki Kawai, Yasushi Kishimoto

**Affiliations:** ^1^Department of Neurological Surgery, Faculty of Medicine, Kagawa University, Miki-cho 761-0793, Japan; ^2^Department of Inflammation Pathology, Faculty of Medicine, Kagawa University, Takamatsu 761-8057, Japan; ^3^Laboratory of Neurobiophysics, Kagawa School of Pharmaceutical Sciences, Tokushima Bunri University, Sanuki 769-2193, Japan; ^4^Kagawa General Rehabilitation Hospital, Takamatsu 761-8057, Japan

## Abstract

There has been growing awareness of the correlation between an episode of traumatic brain injury (TBI) and the development of Alzheimer's disease (AD) later in life. It has been reported that TBI accelerated amyloid-*β* (A*β*) pathology and cognitive decline in the several lines of AD model mice. However, the short-term and long-term effects of TBI by the weight-drop method on amyloid-*β* pathology and cognitive performance are unclear in wild-type (WT) mice. Hence, we examined AD-related histopathological changes and cognitive impairment after TBI in wild-type C57BL6J mice. Five- to seven-month-old WT mice were subjected to either TBI by the weight-drop method or a sham treatment. Seven days after TBI, the WT mice exhibited significantly lower spatial learning than the sham-treated WT mice. However, 28 days after TBI, the cognitive impairment in the TBI-treated WT mice recovered. Correspondingly, while significant amyloid-*β* (A*β*) plaques and amyloid precursor protein (APP) accumulation were observed in the TBI-treated mouse hippocampus 7 days after TBI, the A*β* deposition was no longer apparent 28 days after TBI. Thus, TBI induced transient amyloid-*β* deposition and acute cognitive impairments in the WT mice. The present study suggests that the TBI could be a risk factor for acute cognitive impairment even when genetic and hereditary predispositions are not involved. The system might be useful for evaluating and developing a pharmacological treatment for the acute cognitive deficits.

## 1. Introduction

Traumatic brain injury (TBI) occurs when an external force transmitted to the head results in short- and/or long-term cognitive disability [1]. There has been a growing awareness of the correlation between an episode of TBI and the development of Alzheimer's disease (AD) later in life [2–5], even though several studies have not found a correlation [6, 7]. To elucidate the molecular mechanism by which TBI increases the risk of AD, several studies using mouse models of AD have been performed [8–11]. Most of these studies have found evidence that TBI accelerated the onset of AD-related histopathological changes and cognitive impairment [8, 9].

Recently, we also reported that TBI accelerated amyloid-*β* (A*β*) pathology and cognitive deficits in triple transgenic- (3×Tg-) AD model mice [12, 13]. In the previous study, we used the weight-drop method [14, 15], a closed head injury model, to mimic diffuse axonal injury (DAI) without focal injury to characterize changes that closely parallel the abnormalities characteristic of human diffuse traumatic brain injury that is likely to occur in motor vehicle accidents or falls. In the 3×Tg-AD mice subjected to TBI, the spatial and motor learnings were not significantly different 7 days after TBI compared to those of the sham-treated 3×Tg-AD mice. However, we clearly demonstrated that TBI induces a significant increase in A*β* deposition in the hippocampus compared to control animals 28 days after TBI and this is associated with worse spatial and motor learning abilities in the 3×Tg-AD mice, suggesting that TBI could be a risk factor for accelerated AD progression, particularly when genetic and hereditary predispositions are present. However, the short-term and long-term effects of TBI by the weight-drop method on AD-like pathology and cognitive performance in wild-type (WT) mice are unclear. Hence, the purpose of the present study is to elucidate the effects of TBI on both cognitive function and histopathology in WT mice.

## 2. Materials and Methods

### 2.1. Animals

Behavioral and histological data were obtained from 5- to 7-month-old male wild-type mice on a 129/C57BL6J background, weighing 25–30 g, purchased from CLEA Japan (Tokyo, Japan). The mice were housed in standard acrylic cages with a 12-hour light/dark cycle with light from 8:00 a.m. to 8:00 p.m. All animal procedures were performed in accordance with the guidelines for animal experimentation from the ethical committee of Kagawa University and Tokushima Bunri University.

### 2.2. Traumatic Brain Injury (TBI)

Mice were anesthetized with 2% isoflurane and allowed to breathe spontaneously without tracheal intubation. WT mice were subjected to sham operation or TBI using a weight-drop method to deliver brain injury. However, there are several variations regarding the site of injury and degree of head fixation in previous studies [12–16]. In this study, the mice receiving TBI treatment were placed on a foam-covered platform without head fixation to result in a predominantly diffuse injury induced by a shearing force. A stainless-steel disc helmet (diameter: 5 mm, thickness: 1 mm) was attached using dental cement to the skull vertex (1.5 mm lateral to the midline in the right coronal plane) of each mouse to reduce the risk of skull fractures [12–14]. An iron weight (10 mm in diameter and 30 g in weight) was freely dropped from a height of 80 cm. In order to induce high acceleration upon impact and cause a shearing injury to the brain, the impact area was defined as the right anterior frontal area (1.5 mm lateral to the midline in front of the coronal plane). The mouse was returned to the home cage after a complete recovery from the anesthesia. In the sham-treated control mice, the skull was exposed under anesthesia; the skin incisions were then closed with silk sutures without TBI. TBI or sham treatment was performed in a blinded fashion by another experimenter to that of the behavioral tests.

### 2.3. Behavioral Tests: Morris Water Maze (MWM)

**Figure 1:**
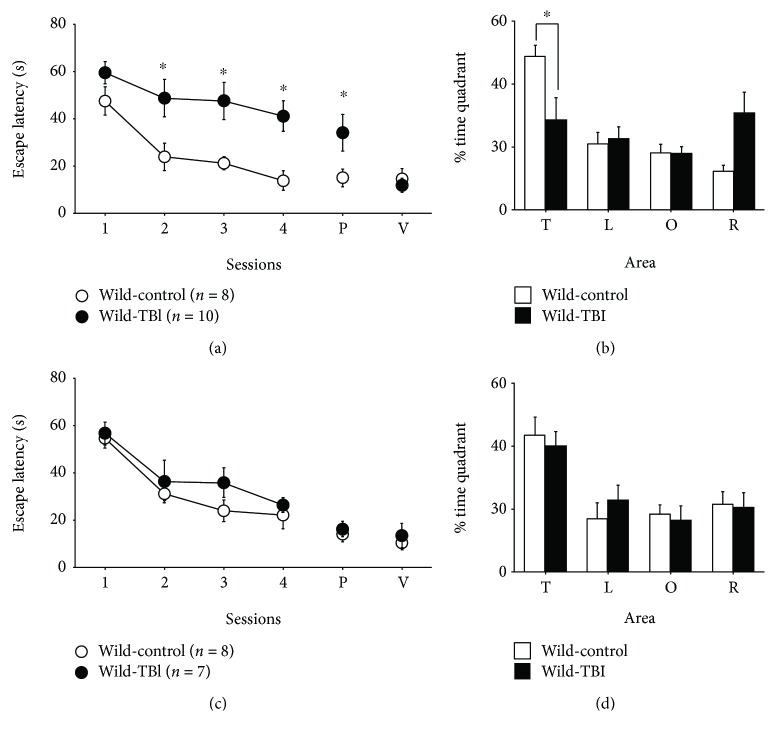
The short- and long-term effects of traumatic brain injury (TBI) on Morris water maze performance in wild-type (WT) mice. (a) The escape latencies in TBI-treated (●, *n* = 8) and sham-treated (○, *n* = 10) WT mice during the training sessions of the hidden-platform task on day 1 (4 days after the injury) through day 4 of training. The probe trial (P) was performed 1 hour after the last trial of the training session on day 4. The visible platform version of the task (V) was carried out 4 days after the injury in an independent preliminary experiment. (b) The percentage of time spent in each quadrant (T: target quadrant; L: left quadrant; O: opposite quadrant; R: right quadrant) during the probe trial on day 4 (7 days after the injury). (c) The escape latencies in TBI (●, *n* = 7) and sham-treated (○, *n* = 8) WT mice during training sessions of the hidden-platform task on day 1 (25 days after the injury) through day 4. The probe trial (P) was performed 1 hour after the last trial of the training session on day 4. A visible platform version of the task (V) was carried out 32 days after the injury in an independent preliminary experiment. (d) The percentage of time spent in each quadrant during the probe trial on day 4 (28 days after the injury). ^∗^*p* < 0.05, when compared with sham-treated WT mice.

MWM was performed as described previously [17–21]. The water maze pool (Eiko Science, Tokushima, Japan), which had a diameter of 120 cm, contained opaque white water (24 ± 2°C) with a translucent platform (diameter, 10 cm) that was submerged 1 cm below the surface. Four sheets of paper with black and white geometric designs were attached to the walls of the experimental room as additional cues. After pretraining (8 trials per day for 2 days), the MWM test was started at either 4 or 25 days after TBI or sham treatment in the WT mice. Mice were only used in one of the two MWM experiments to evaluate the short- and long-term effects of TB; a different set of mice were used for the results shown in Figures 1(a) and 1(c) and Figures 1(b) and 1(d). The hidden-platform task took 4 days to perform (4 sessions per day, at least 1 hour apart). Mice that failed to reach the platform within 80 s were led to the platform by placing a finger. Their performance was monitored and analyzed with an automated video-tracking system (TopScan, CleverSys Inc., Reston, VA, USA). The behavioral data for one mouse was excluded from further MWM analysis due to a failure of the movie tracking system.

### 2.4. Histopathological Study

The method employed was the same as that described previously [12, 13]. After performing the MWM test 7 days or 28 days after TBI or sham-TBI treatment, the WT mice were euthanized to remove the brain. The brains were postfixed in 4% formaldehyde and immersed in 30% sucrose in 0.1 M cold phosphate-buffered saline (pH 7.4) and embedded and sliced into 4 *μ*m thick coronal sections. To clarify the location of A*β* deposits in the hippocampus, hematoxylin and eosin (H&E) staining was performed. The distribution of A*β* deposits was examined using immunostaining with 4G8 anti-A*β*17-24 monoclonal antibody (1 : 1000; Covance, Princeton, NJ). The APP accumulations were examined by immunostaining using anti-APP polyclonal antibody (1 : 1000; AnaSpec, Fremont, CA). The secondary detection methods used to detect the primary antibody are described in a previous report [13]. Light microscope images of the hippocampus were captured from a series of five to seven sections using a microscope (ECLIPS Ci; Nikon, Tokyo, Japan) with a 4x objective lens. Outlining the region of interest according to a brain atlas and H&E staining helped us to confirm the same region of the hippocampus in each sample. Each image was analyzed using image analysis software (ImageJ from NIH). The areas occupied by APP- or A*β*-immunoreactive products in the hippocampus were measured, and the total area occupied by the outlined structures was measured to calculate the percentage of the area occupied by immunoreactive products over the total outlined anatomical area in the image [22]. For APP quantification, only a part of the hippocampal CA1 area was selected. In contrast, the total hippocampal area was used for A*β* quantification. APP/A*β*-positive areas in each region averaged between 5 and 7 sections with about 100 *μ*m spacing per mouse and from regions that fell within −1.40 and −2.20 mm to the bregma. The quantification was performed only on the ipsilateral side to TBI. We randomly selected the animal for A*β* and/or APP immunostainings from the mice that were analyzed in the behavioral tests.

### 2.5. Statistical Analysis

The histopathological data were analyzed using Student's or Welch's two-tailed *t*-test. The MWM data were analyzed by two-way repeated measure ANOVA with Bonferroni post hoc test or Student's two-tailed *t*-test. All the data are presented as mean ± SEM. *p* < 0.05 was considered statistically significant.

## 3. Results

### 3.1. Behavioral Analysis: Short- and Long-Term Effects of TBI on Cognitive Impairment in WT Mice

First, to examine the acute effect of TBI on cognitive function in WT mice, we used the Morris water maze (MWM) to assess 5- to 7-month-old WT mice from 4 to 7 days after TBI. The escape latencies in both TBI- and sham-treated (control) WT mice decreased during the training, indicating memory acquisition. However, a significant difference was observed between the two groups, with a longer escape latency in TBI-treated mice than in sham-treated mice (*F*_(1, 16)_ = 13.18 and *p* = 0.0022 for group effect; *F*_(3, 48)_ = 0.99 and *p* = 0.40 for interaction effect; Figure 1(a)). After the acquisition phase, the platform was removed and the probe trial (P) was performed using sham-treated and TBI-treated mice. The latency to reach the site where the platform had been placed was significantly longer in TBI-treated mice than in control mice (Figure 1(a), *p* < 0.05), and the percentage of time spent in the target quadrant (T) was significantly less in TBI-treated mice (Figure 1(b), *p* = 0.024). Next, in order to examine the visual and motor deficits in TBI-treated mice, the mice were allowed to run in the visible platform version of the MWM. There were no differences in the latencies for reaching the visible platform between the two groups (Figure 1(a), *p* > 0.05). The distances of the swimming paths of the TBI-treated WT mice exhibited a similar tendency as the results of the latency analysis (data not shown), indicating that the TBI-treated WT mice swam significantly longer distances until they reached the site where the platform had been placed during the training trial compared with the sham-control group. Swimming speeds were not significantly different between the control and TBI group (data not shown). Taken together, these results indicated that spatial learning ability was impaired in WT mice just after TBI treatment.

Next, we examined the long-term effect of TBI on cognitive function in WT mice by testing mice in the MWM at 25 to 28 days after TBI (Figures 1(c) and 1(d)). The escape latency to reach the platform was significantly decreased in both groups and no significant differences were observed between the two groups (*F*_(1, 13)_ = 1.37 and *p* = 0.26 for group effect; *F*_(3, 39)_ = 0.43 and *p* = 0.73 for interaction effect; Figure 1(c)). In the probe trial (P), the latency to reach the site where the platform had been placed was not also significantly different between the TBI- and sham-treated groups (Figure 1(c)), and the percentage of time spent in the target quadrant (T) was not significantly different between the two groups (Figure 1(d)). The swimming path length to the site where the platform had been placed was not significantly different between TBI-treated and control mice (data not shown). These results indicate that spatial learning ability recovered in TBI-treated WT mice by the 28th day after TBI.

### 3.2. Histopathology

#### 3.2.1. APP Accumulation

**Figure 2:**
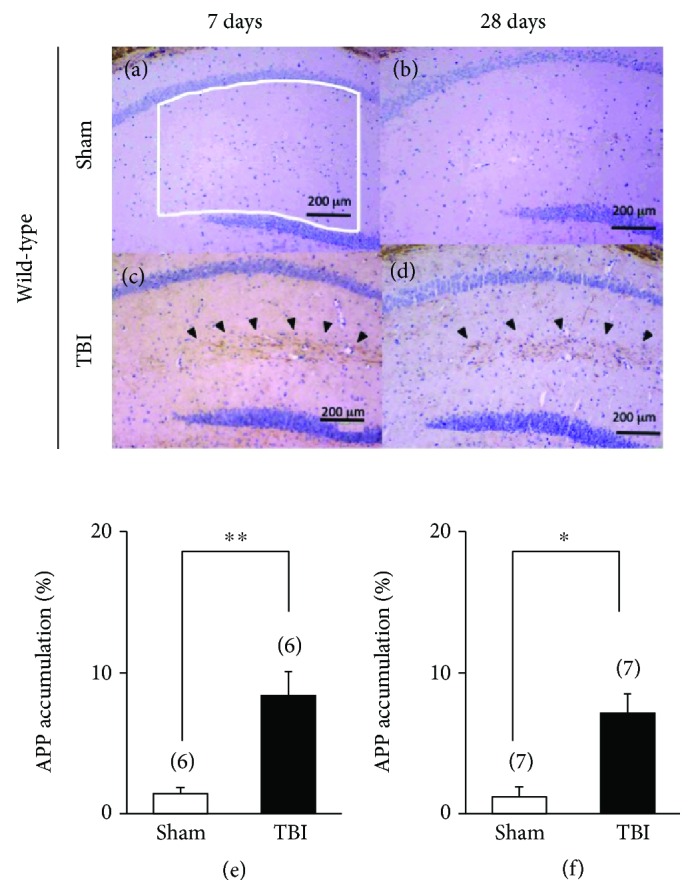
Short-term and long-term effects of traumatic brain injury (TBI) on immunohistochemistry of amyloid precursor protein (APP) accumulation in wild-type (WT) mice. (a, b) Representative photographs demonstrating the axonal immunoreactivity for APP in the hippocampal commissure 7 days after the injury: (a) sham-treated WT mice and (b) TBI-treated WT mice. (c, d) Representative photographs showing the axonal immunoreactivity for APP in the hippocampal commissure 28 days after the injury: (c) sham-treated WT mice and (d) TBI-treated WT mice. Arrowheads represent APP-positive areas in the hippocampus. (e, f) Partial quantification of hippocampal APP accumulation. The presence of APP (expressed as the percentage of the area occupied by APP-immunopositive deposition in the ipsilateral hippocampus) was assessed. The region of CA1 used for APP quantification was outlined by a white line in (a). APP accumulation was significantly greater in the TBI-treated WT (closed bar) mouse hippocampus both 7 days and 28 days after injury. ^∗∗^*p* < 0.01 and ^∗^*p* < 0.05 when compared with sham-treated WT mice (open bar). Scale bar, 200 *μ*m.

After the MWM, we evaluated AD-related histopathological changes after sham treatment or TBI in the WT mice. In sham-treated WT mice, no amyloid precursor protein- (APP-) positive cells were found in the hippocampus at any time (Figures 2(a) and 2(c)). In contrast, positive APP immunostaining in the injured axon was observed in the hippocampal commissure 7 days after TBI (Figure 2(b)), and a few positive APP immunostainings were also seen in the subjacent cortical lesions including the cingulate cortex in WT mice. Furthermore, 28 days after injury, the positive APP immunohistochemical expressions remain in the hippocampal commissure (Figure 2(d)). Statistical analysis indicated that APP accumulation was significantly higher in TBI-treated mice than in sham-treated mice both 7 days and 28 days after injury (Figures 2(e) and 2(f), *p* < 0.0001, *p* = 0.012, respectively). These results indicate that TBI induces axonal damage resulting in APP accumulations in the hippocampus and the accumulations progress even in WT mice.

#### 3.2.2. Amyloid-*β* Deposition

**Figure 3:**
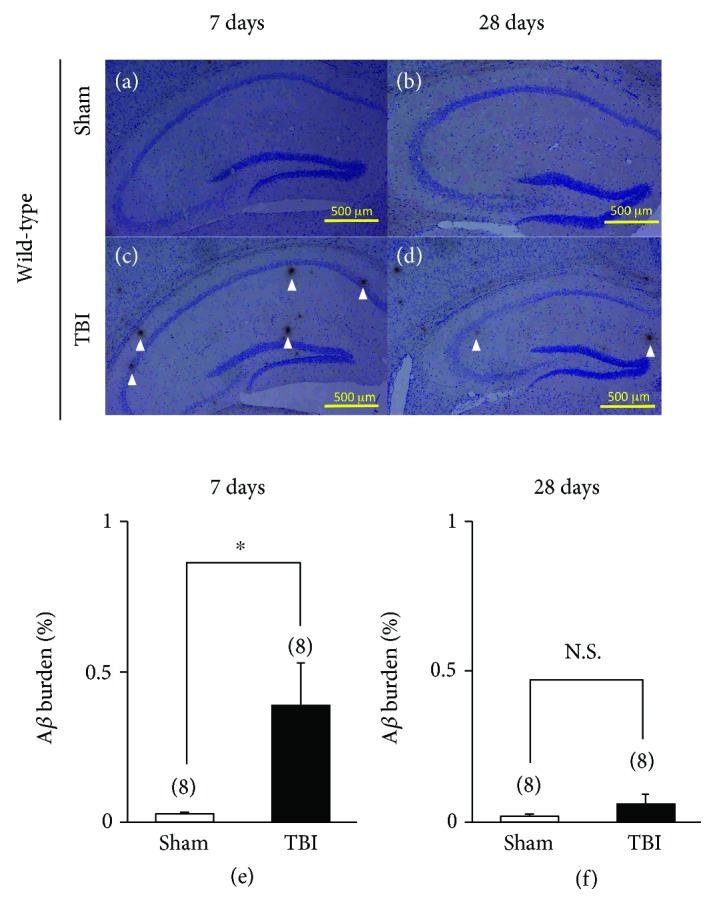
Short-term and long-term effects of traumatic brain injury (TBI) on immunohistochemistry of amyloid-*β* (A*β*) in wild-type (WT) mice. (a, b) Representative photographs demonstrating the axonal immunoreactivity for A*β* in the hippocampus 7 days after the injury: (a) sham-treated WT mice and (b) TBI-treated WT mice. (c, d) Representative photographs showing the axonal immunoreactivity for A*β* in the hippocampus 28 days after the injury: (c) sham-treated WT mice and (d) TBI-treated WT mice. Scale bar, 500 *μ*m. Arrowheads represent A*β* deposits in the hippocampus. (e, f) Partial quantification of the hippocampal A*β* deposition. The presence of A*β* (expressed as the percentage of the area occupied by A*β*-immunopositive deposition in the ipsilateral hippocampus) was assessed. (e) WT mice 7 days after sham (control) operation (*n* = 8) or the injury (*n* = 8). (f) WT mice 28 days after sham (control) operation (*n* = 8) or the injury (*n* = 8). N.S.: not significant; ^∗^*p* < 0.05, when compared with sham-treated WT mice. N.S. scale bar, 500 *μ*m.

In WT mice, no extracellular A*β* deposition was observed in the brain at either 7 or 28 days after the sham operation (Figures 3(a) and 3(c)). On the other hand, in TBI-treated WT mice, several A*β* depositions were observed 7 days after the injury in the hippocampal CA3 and the dentate hilus (Figure 3(b)). However, the burden and distribution of A*β* depositions in the hippocampus was scattered and decreased 28 days after injury in TBI-treated WT mice (Figure 3(d)).

To quantitatively determine the effects of TBI on progressive A*β* deposition, the area occupied by A*β*-immunopositive deposition in the ipsilateral hippocampus was assessed using an image analysis system. The burden of A*β* was significantly higher in TBI-treated mice compared to sham-treated mice 7 days after the injury (Figure 3(e), *p* = 0.022). However, the burden of TBI-induced A*β* deposition was significantly decreased, and only a trace level of A*β* deposition was observed 28 days after the injury (Figure 3(f), *p* = 0.29).

## 4. Discussion

In the present study, we demonstrated that TBI induced short-term cognitive deficits in WT mice; however, the deficits were largely recovered 28 days after the injury. Correspondingly, A*β* deposits were transiently expressed in the WT mouse hippocampus 7 days after TBI.

TBI has devastating acute effects and induces subsequent long-term neurodegeneration. Acute or chronic learning impairment has been reported in the existing models of concussive brain injury in the wild-type rodents [23–25]. However, our histopathological results are obviously inconsistent with previous studies that found that APP and IL-1*β* are rapidly elevated, but no A*β* deposits were observed just after TBI [26, 27]. These previous studies used moderate to severe levels of injury induced by controlled cortical impact [8, 26, 27] or the fluid-percussion model [28, 29]. The weight-drop method used in the present study is a closed head injury model used to mimic diffuse axonal injury (DAI), resulting in a predominantly diffuse injury from shearing force [16]. Therefore, the discrepancy between these findings on A*β* deposits might at least be partially due to the difference in TBI methods used. Indeed, Tian et al. reported that A*β* was significantly elevated in wild-type Sprague-Dawley rats 1, 7, or 14 days after TBI induced by the weight-drop method although they did not test A*β* deposition 28 days after injury [30]. Regardless, the other differences, such as strain of mice/rats, the age tested, the duration between TBI and histological analysis, the impact site (lateral or midline injury), and/or their complex interactions may also affect the degree of A*β* deposits in naive animals.

The use of immunohistology to assess changes in APP in axons has been used as a sensitive marker for axonal injury [31, 32]. In our previous study, we reported that TBI induced early APP accumulation and a few A*β* deposits were observed in the hippocampus in 3×Tg-AD mice within 7 days of the injury. Considering the previous study [12, 13], TBI induced early APP accumulations and scattered A*β* depositions in the hippocampus both in WT and 3×Tg-AD mice 7 days after injury. However, there is a distinct difference in the subsequent courses of A*β* pathology in the hippocampus and cognitive impairment between WT and 3×Tg-AD mice. TBI-treated WT mice showed several injured axons stained for APP and scattered A*β* depositions in the hippocampus 7 days after TBI, and the animals showed lower spatial learning disability compared to control mice. These acute cognitive and histological impairments were ameliorated by 28 days after the injury, and TBI-treated WT mice showed similar spatial learning and A*β* deposition in the hippocampus to control mice. This temporal pattern of A*β* deposition in the traumatized brains observed in TBI-treated WT mice is consistent with that observed in human pathological studies [33–35] and in our recent amyloid positron emission tomography (PET) study of patients with post-TBI neuropsychological impairment, in which A*β* deposition was negative in many of the patients at the chronic stage after TBI (5–129 months, median 54 months) [36].

The burden of A*β* deposition and learning disability is milder in WT mice than in 3×Tg-AD mice at a chronic time after the injury [12]. This is in accordance with the result of a recent population-based amyloid PET study in humans which showed that self-reported head trauma is associated with greater A*β* deposition in individuals with mild cognitive impairment (MCI), but not in cognitively normal individuals [37]. These findings suggest that TBI may accelerate AD-related pathology in individuals who have innate A*β* pathology in the brain, but not in the healthy brain. The recognition of a cause and effect link between TBI and AD pathology aids in enlightening the importance of the prevention of head trauma, especially in elderly people. Moreover, the results can promote the development of treatment strategies that block the adverse effects of AD-related pathophysiological processes after acute TBI.

Several limitations of this study should be acknowledged. First, we did not quantify three-dimensional A*β* deposition in the brain. However, the ImageJ software from NIH is a useful tool for the semiquantification of microscopic images. Indeed, previous reports evaluated A*β* plaque density using the same method and showed reasonable results [33, 38]. This method is very simple for A*β* plaque analysis by outlining the region of interest (ROI) and measurement of number for the total area occupied by the immunostaining in the ROI of the sections. Second, the histological evaluation was performed only in the hippocampus, and neither the cortical layer nor the basal ganglia were examined. While the cortex is directly implicated in TBI, secondary cell death is ensured in the hippocampus, a critical brain structure for learning and memory. As the hippocampus is a well-established neurogenic site highly sensitive to both acute and chronic injury, this specific brain region appears to be an optimal site to study secondary pathological disturbance after TBI [39]. Third, we did not evaluate the relationship between tau protein accumulation and cognitive impairment in the present and previous studies [12, 13]. In the mouse model, A*β* pathology appears to be upstream of tau pathology. For example, intracerebral injection of aggregated A*β* increased tau phosphorylation and therefore the numbers of neurofibrillary tangles (NFTs) in both local and distant regions in transgenic mice [40]. On the other hand, human pathological studies suggest that A*β* and tau pathologies may be independent in the context of TBI or at least that the relationship may be more complex. In repetitive concussive TBI resulting in chronic traumatic encephalopathy, 100% of cases had widespread NFTs but a smaller subset had A*β* pathology [41]. Further studies are needed in order to elucidate these unresolved issues.

## 5. Conclusions

The results of the behavioral and histopathological investigations presented in the current study demonstrate that TBI induces a transient increase in A*β* deposition in the hippocampus relative to control animals 7 days after TBI and this parallels acute cognitive impairment even in naive wild-type mice. Considering and compared with our previous data on the 3×Tg-AD mice [12, 13], the present data might provide evidence of a close interrelation between A*β* and cognitive decline, and the presence of an innate A*β* clearance mechanism in WT mice, but not in 3×Tg-AD mice. The system presented in this study might be useful for evaluating and developing a pharmacological treatment for the acute cognitive impairment.

## Data Availability

The data used to support the findings of this study are available from the corresponding author upon request.
